# Experimental hut and field evaluation of a metofluthrin-based spatial repellent against pyrethroid-resistant *Anopheles funestus* in Siaya County, western Kenya

**DOI:** 10.1186/s13071-023-06096-2

**Published:** 2024-01-04

**Authors:** Silas Agumba, Vincent Moshi, Margaret Muchoki, Seline Omondi, Jackline Kosgei, Edward D. Walker, Bernard Abong’o, Nicole Achee, John Grieco, Eric Ochomo

**Affiliations:** 1 Centre for Global Health Research, Kenya Medical Research Institute, P.O. Box 1578-40100, Kisumu, Kenya; 2 Department of Microbiology and Molecular Genetics, Michigan State University, East Lansing, MI 48824 USA; 3 Department of Biological Sciences, Eck Institute for Global Health, University of Notre Dame, Notre Dame, IN USA

**Keywords:** *Anopheles funestus*, Emanator, Metofluthrin, Spatial repellent, Western Kenya

## Abstract

**Background:**

Spatial repellents (SR) may complement current vector control tools and provide additional coverage when people are not under their bednets or are outdoors. Here we assessed the efficacy of a metofluthrin-based SR in reducing exposure to pyrethroid-resistant *Anopheles funestus* in Siaya County, western Kenya.

**Methods:**

Metofluthrin was vaporized using an emanator configured to a liquid petroleum gas (LPG) canister, placed inside experimental huts (phase 1) or outdoors (phase 2), and evaluated for reductions in human landing rate, density, knockdown and mortality rates of *An. funestus*, which are present in high density in the area. To demonstrate the mosquito recruiting effect of LPG, a hut with only an LPG cooker but no metofluthrin was added as a comparator and compared with an LPG cooker burning alongside the emanator and a third hut with no LPG cooker as control. Phase 2 evaluated the protective range of the SR product while emanating from the centre of a team of mosquito collectors sitting outdoors in north, south, east and west directions at 5, 10 and 20 feet from the emanating device.

**Results:**

Combustion of LPG with a cook stove increased the density of *An. funestus* indoors by 51% over controls with no cook stove. In contrast, huts with metofluthrin vaporized with LPG combustion had lower indoor density of *An. funestus* (99.3% less than controls), with knockdown and mortality rates of 95.5 and 87.7%, respectively, in the mosquitoes collected in the treated huts. In the outdoor study (phase 2), the outdoor landing rate was significantly lower at 5 and 10 feet than at 20 feet from the emanator.

**Conclusions:**

Vaporized metofluthrin almost completely prevented *An. funestus* landing indoors and led to 10 times lower landing rates within 10 feet of the emanator outdoors, the first product to demonstrate such potential. Cooking with LPG inside the house could increase exposure to *Anopheles* mosquito bites, but the use of the metofluthrin canister eliminates this risk.

**Graphical abstract:**

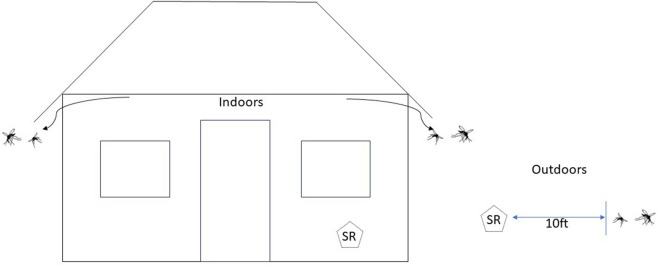

## Background

In 2021, 247 million cases of malaria and 619,000 deaths were recorded globally [1]. The main tools recommended for public health malaria vector control are long-lasting insecticidal nets (LLINs) and indoor residual spraying (IRS), which have led to a massive reduction in cases across endemic countries [2]. Despite the efforts at expansive coverage with these tools, malaria persists, with a disproportionate burden in sub-Saharan Africa (SSA) owing to the mix of a set of efficient *Anopheles* species and *Plasmodium falciparum* [1] and the emergence and spread of insecticide resistance, among other factors [3]. In addition, local vector species may exhibit insecticide avoidance, so that mosquitoes actively avoid treated surfaces, adopting a more outdoor biting and resting behaviour and resorting to feeding on animals [4].

In view of these challenges, additional approaches and interventions are required to make further progress in the fight against malaria [5]. Indoor deployment of LLINs and IRS means that they cannot directly protect people outdoors, apart from the benefit derived from a community-wide reduction in mosquito density, and they are also challenging to implement where people do not have permanent homes, such as immigrant or nomad communities. Additionally, IRS is expensive to scale up, hence limiting its coverage to the population at risk [6]. Therefore, vector control measures that can target gaps in protection such as early evening, late morning and outdoor transmission as well as mobile populations are highly desirable. Intra-domiciliary measures are insufficient to reach elimination, and there are calls for new and innovative tools for malaria vector control [7]. Spatial repellents (SR) are a promising vector control paradigm that could add to the existing strategies for malaria prevention [8, 9]. SRs such as mosquito coils have been shown to reduce mosquito biting [10, 11] in studies conducted in Indonesia [11], Peru and China [12]. Additional evidence is currently being generated to show the efficacy of SRs across a range of malaria transmission endemicity characteristics and mosquito vector species and in the context of high LIIN coverage before SRs can be recommended as a tool for malaria control by national malaria control programmes (NMCPs) [13, 14].

SRs have many potential advantages over existing malaria control tools. In contrast to insecticide-treated nets, SRs placed within a house may protect all residents, and in particular at times when they are not under LLINs. SRs with a residual effect would not require daily placement and monitoring like IRS, and would reduce user bias. Unlike IRS, SRs would be effective against mosquitoes that have adapted to avoid landing on treated surfaces within the household. There are very few tools such as attractive targeted sugar bait that have shown efficacy against outdoor biting mosquitoes, especially in rural African settings where malaria is rife [15]. Metofluthrin, as a promising SR, acts through the disruption in orientation towards the host (preventing bites) and knockdown and killing of mosquitoes [16]. SRs have been formulated as coils, paper, gel, liquid and other types of emanators which can be either active (requiring a source of heat) or passive (not requiring a source of heat) [10]. The product used for these evaluations has been assessed for human safety and exposure and is now available in a formulation that is released slowly when heated [17, 18]. Liquefied petroleum gas (LPG)-activated metofluthrin-based SRs have recently been developed by Thermacell Repellents, Inc. (Bedford, MA, USA). LPG is quickly being adopted for cooking in multiple parts of rural and urban Africa [5, 19, 20]. Cooking with LPG in experimental huts in Rwanda increased *Anopheles* density relative to huts where traditional cooking fuels such as wood or charcoal were burned because burning LPG produces carbon dioxide as a by-product, thus creating an attractant for host-seeking mosquitoes [21].

This study evaluated the efficacy of metofluthrin cartridges attached to LPG-based cookers against malaria vectors in reducing mosquito entry into experimental huts in western Kenya, with major outcomes being reductions in landing rates, deterrence and induced exophily. The study also evaluated the minimum optimal duration of emanation of the SRs required to achieve overnight efficacy in experimental huts as well as the distance of protection realized from outdoor placement of the SRs in the rural village compounds. In addition, the study evaluated the role of cooking with LPG in recruiting *Anopheles* mosquitoes indoors relative to huts without LPG cooking, and whether volatilization of metofluthrin indoors, where LPG cooking takes place, obviated the attractive effect of combusted LPG to *Anopheles* mosquitoes.

## Methods

### Study site

The study was conducted in the *Dala Suna* experimental hut site on the shores of Lake Kanyaboli (0° 02′ 08.5″ N, 34° 11′ 05.0″ E) and in villages close to the hut site in Alego-Usonga sub-County, Siaya County, western Kenya. It is situated close to the swamps that provide conducive breeding habitats for malaria vectors, and characterized by high year-round abundance of *Anopheles funestus* and seasonal peaks of *Anopheles arabiensis*, with average household density > 300 and > 20 per night, respectively (Ochomo et al., unpublished). The area experiences two rainy seasons, one from March to May and the other from October to November, with high malaria transmission occurring throughout the year [22]. The primary economic activities of the local population are subsistence farming, livestock keeping, fishing and small-scale trading.

### Experimental huts

**Fig. 1:**
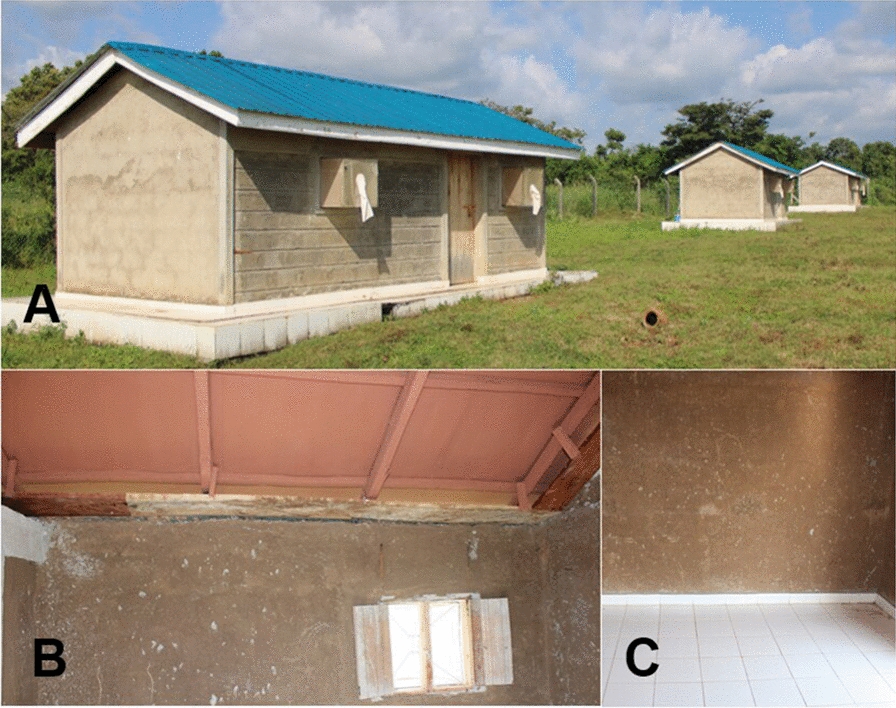
Experimental hut design: **A** front view of the hut fitted with window exit traps, **B** showing the wood baffles, and **C** showing the tiled floor and the hut interior walls

There are seven experimental huts, each measuring 6 m long, 3 m wide and 2 m high. The experimental huts are designed to resemble a typical Kenyan household in terms of structure and mosquito exit/entry points (eaves, windows and doors) (Fig. 1a). Mosquito exit traps were fitted to all four windows of the experimental huts—two windows on the front face and two on the backside of the huts. The walls are made of blocks and lined with mud on the inside. The huts have corrugated iron roofs and a 10-cm eave gap on all sides. To prevent mosquitoes from exiting the huts, baffles are installed at the eave gaps, allowing easy entry for mosquitoes (Fig. 1b). In addition, the floors are tiled with white tiles for ease of collection of knocked-down and dead mosquitoes (Fig. 1c). The huts are elevated above the ground on a concrete base surrounded by a water-filled moat to keep ants away [23].

### Experimental design and set-up

**Fig. 2:**
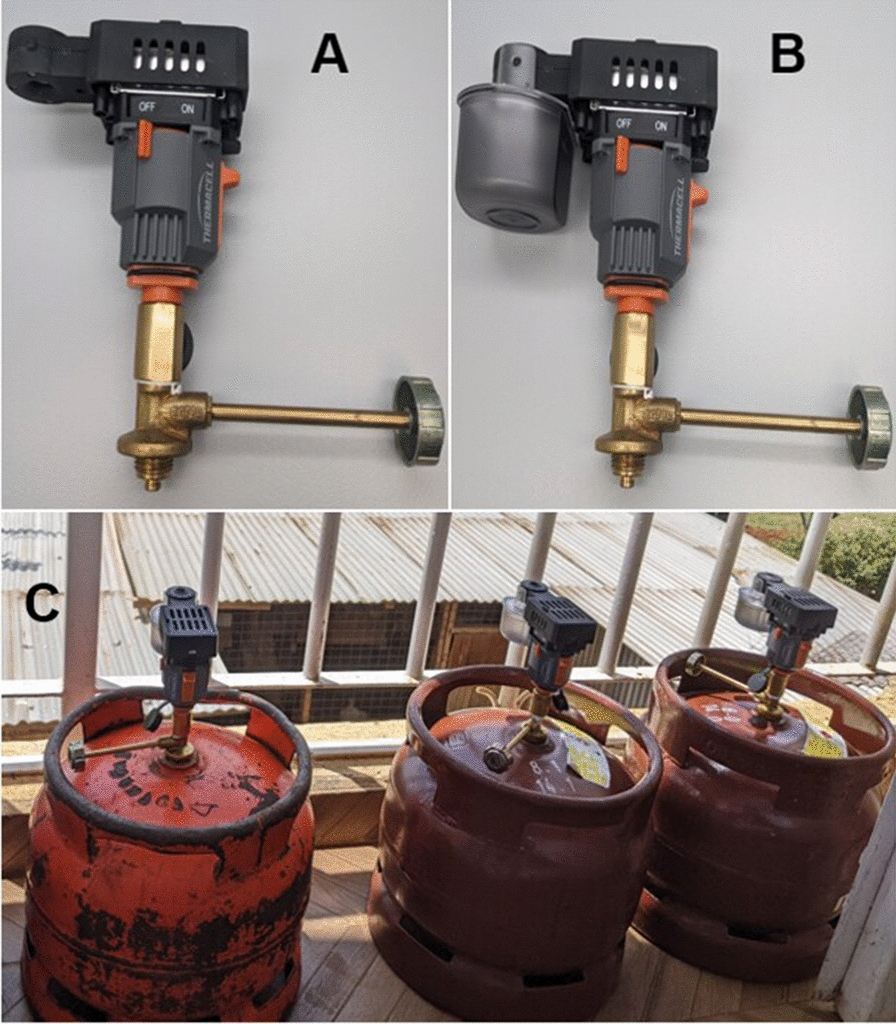
**A** Thermacell emanator, **B** metofluthrin cartridge attached to the Thermacell emanator–metofluthrin SR container, **C** Thermacell emanator attached to 6-kg gas cylinders

The trial was conducted in two phases, with phase 1 evaluations being carried out indoors in experimental huts and phase 2 evaluations conducted outdoors in compounds in the nearby village. The study used two collection methods to assess the effectiveness of the emanator in preventing mosquitoes from landing on study participants and entering the huts. Human landing catch (HLC) was used to determine the landing rates of mosquitoes on humans [23], both indoors and outdoors, while mouth aspiration was used to determine indoor resting density as a measure of the deterrence and induction of exophily by the emanator. Volunteers aged between 18 and 45 years from neighbouring villages who consented to participate were recruited, trained and tested for malaria. Those who tested positive were treated with antimalarial medication. All the study participants were placed on prophylaxis with a weekly dose of Mefloquine^®^ once clear of infection or if they had a negative test at the time of consenting. The SR device manufactured by Thermacell Repellents, Inc. (Bedford, MA, USA) vaporizes metofluthrin as its active ingredient. It functions like a diffuser by using heat to evaporate a small amount of insecticide into the air. The canister containing metofluthrin is attached to the LPG cylinder. Once the gas burner is turned on, it produces heat that vaporizes the metofluthrin SR into the air. The complete set of Thermacell technology, along with the 6-kg cylinder gas used during emanation is shown in Fig. 2, where the Thermacell emanator consists of three main parts. Part A is the Thermacell technology that attaches to the gas cylinder. Part B is where the metofluthrin SR container/cartridge is held in position when in use. Part C shows the metofluthrin cartridge fixed into a 6-kg gas cylinder during emanation.

### Human landing catch

HLC was conducted for 12 h (18:00–06:00) every night for five nights inside the experimental huts as shown in Table 1. Each study participant collected mosquitoes throughout a 6-h shift (18:00–00:00 or 00:00–06:00) with a 15-min break within every hour of collection and were rotated between the shifts. During the mosquito collection process, the collectors sat in a chair wearing shorts and long-sleeved shirts. They used a mouth aspirator (Model 412, John W. Hock Company, Gainesville, FL, USA) to collect the mosquitoes that landed on their lower legs. Any mosquitoes that were collected were placed in a clean paper cup. These cups were changed every hour and for each location. The mosquitoes were provided access to 10% sugar solution. At the end of each night collection, the mosquitoes were transported in a cooler box to the field laboratory for processing. The paper cups were sorted, and those with mosquitoes were placed in the killing jar with chloroform soaked in cotton wool to knock down mosquitoes. The mosquitoes were sorted, the culicines were counted, and the number of males and females recorded and then discarded. The *Anopheles* mosquitoes were separated by species, sex and abdominal status (blood-fed, non-blood-fed or gravid) for females, and numbers collected per hour were recorded. Morphological identification was performed on the mosquitoes using taxonomic keys [24] to differentiate between *An. funestus* sensu lato (s.l.) and *Anopheles gambiae* s.l. and other secondary malaria vectors.

**Table 1:** Schedule of experiments conducted in the experimental huts including the rotation of the sleepers and HLC volunteers

Date	Activity	Hut 1	Hut 2	Hut 3
23-Aug-22	Sleepers	3	2	6
24-Aug-22	HLC	2 and 3	1 and 4	5 and 6
25-Aug-22	HLC	4 and 1	6 and 5	3 and 2
26-Aug-22	Sleepers	2	6	3
28-Aug-22	Sleepers	6	3	2
29-Aug-22	HLC	5 and 6	2 and 3	1 and 4
30-Aug-22	HLC	3 and 2	4 and 1	6 and 5
31-Aug-22	HLC	1 and 4	5 and 6	2 and 3
01-Sept-22	Sleepers	3	2	6
02-Sept-22	Sleepers	2	6	3

### Aspiration collections

Aspiration collections were carried out in the morning following overnight sleeping in the experimental huts. The participants reported sleeping in the huts under an untreated bednet from 20:30 until 06:30. Following World Health Organization (WHO) guidelines on simulating wear and tear, each net was intentionally holed with six 16-cm^2^ holes (two holes on each long side and one hole on each short side) [25]. Trained field assistants conducted mosquito collections using mouth aspirators every morning from 06:30 to 07:30 after overnight sleeping activity. Mosquitoes were scored by location—net and under bed, roof, floor, wall and exit traps—and transported in a cooler box to the field laboratory for sorting as dead or alive, unfed, fed, gravid or half-gravid. Knockdown and dead mosquitoes were recorded 1 h post-collection. Live mosquitoes were held at 27 ± 2 °C and provided access to 10% sugar solution for up to 24 h to assess delayed mortality. Females were identified at the species level using morphological keys [24].

### Experimental hut evaluation to assess the impact of LPG use on mosquito activity

Phase 1 of the study was conducted for 10 nights from 23 August to 2 September 2022 to investigate whether cooking with LPG gas impacted mosquito activity in three experimental huts: Hut 1 was allocated an LPG cooker burning from 18:00 to 20:00 to simulate local cooking practices. Hut 2 had an LPG cooker burning for 2 h (18:00–20:00), and a metofluthrin emanator attached to a separate LPG cylinder ran for 12 h from 18:00 to 06:00. The third hut was a negative control with neither a metofluthrin emanator nor an LPG cooker. Aspiration and HLC were used for mosquito collections for five alternate nights each (Table 1).

### Evaluation of the residual efficacy of the SR emanator to inform combination deployment with LLINs

Between January and February 2023, the efficacy of the SR product deployed for limited durations was evaluated in comparison with an all-night deployment to evaluate any residual efficacy and to inform possible deployment in local households with optimal coverage and use of LLINs. Four different durations of metofluthrin emanation—0 h (control), 2 h (18:00–20:00), 4 h (18:00–22:00) and 12 h (18:00–06:00)—were evaluated. To understand the efficacy in reducing morning biting, additional emanation for 1 h was added to the 2-h and 4-h arms between 05:00 and 06:00.

### Small-scale field trial to determine outdoor protective efficacy

The field trial was conducted to test the effectiveness of the metofluthrin emanator outdoors. The trial involved 10 compounds for each of the emanation distances of 5, 10 and 20 feet. In each compound, we placed the SR emanator attached to the LPG cylinder at the centre and had four HLC volunteers stationed equidistantly in the north, south, west and east directions from the emanator. Volunteers were paired up and rotated every night, each working a 6-h shift from either 18:00 to 00:00 or 00:00 to 06:00, and each volunteer pair rotated through the four directions. To prevent exhaustion, volunteers took a break after three consecutive nights of HLC. The emanators were run continuously from 18:00 to 06:00.

### Statistical analysis

All the data collected were entered into the Commcare^®^ version 2.53.1 platform and parameters such as vector abundance assessed using descriptive statistics (means, proportions and 95% confidence intervals [CI]). Generalized linear mixed models (GLMM) using Template Model Builder (package glmmTMB) were fitted using negative binomial distribution for analysis of mosquito numbers at different emanation periods. Models were adjusted for repeated measures using the hut or compound ID and hour as random effects. All data analyses were performed using R statistical software version 4.1.2, and the significance level was set at α = 0.05.

## Results

### Species composition

During the study period, a total of 3995 mosquitoes were collected. Among all the *Anopheles* collected, the most abundant species was *An. funestus*, with a total of 2547, constituting 100% of the *Anopheles* population, followed by *An. arabiensis* with only 11 mosquitoes, representing 0%. In addition, a total of 1437 *Culex* spp. mosquitoes were collected. It is worth noting that only *An. funestus* is reported in these results, as it was the most abundant species of *Anopheles* collected.

### Hut entry

Higher average numbers of *An. funestus* mosquitoes were recorded in the hut with LPG (*N* = 302.3) than in the control hut (*N* = 199.6) and the hut with metofluthrin and LPG (*N* = 36) (Table 2). Of mosquitoes that entered the hut, the metofluthrin SR knocked down 95.5% of the mosquitoes collected in the treated hut, resulting in 87.7% mortality after 24 h. The use of LPG increased *An. funestus* mosquito entry by 51%; however, indoor use of metofluthrin SR deterred *An. funestus* mosquito entry by 82%.

**Table 2:** Mean density of *Anopheles* mosquitoes caught in the experimental huts during the evaluation of whether cooking with LPG gas has an impact on mosquito activity inside a house

Mosquitoes collected	Collection location/point	Hut 1 (LPG only)	Hut 2 (metofluthrin + LPG)	Hut 3 (control)
*An. funestus* (female)	Floor	0	19.4	0
	Net	33.4	0	16.8
	Roof	93.4	0	53.4
	Wall	18.2	0	14.4
*An. funestus* (male)	Floor	0	13	0
	Net	42.2	0	17.6
	Roof	82.6	0	62.2
	Wall	15.8	0	20.4
*An. gambiae* (female)	Floor	0	1.2	0.2
	Net	3	0	4
	Roof	4.8	0	2.8
	Wall	1.4	0	1
*An. gambiae* (male)	Floor	0	2.2	0.8
	Net	3.2	0	2.8
	Roof	3	0	1.8
	Wall	1.2	0	1.4
Average *An. funestus* entry per hut		302.2	35.8	199.6
Percentage of total collected per hut		56.2	6.7	37.1

### Metofluthrin efficacy in the experimental hut

**Fig. 3:**
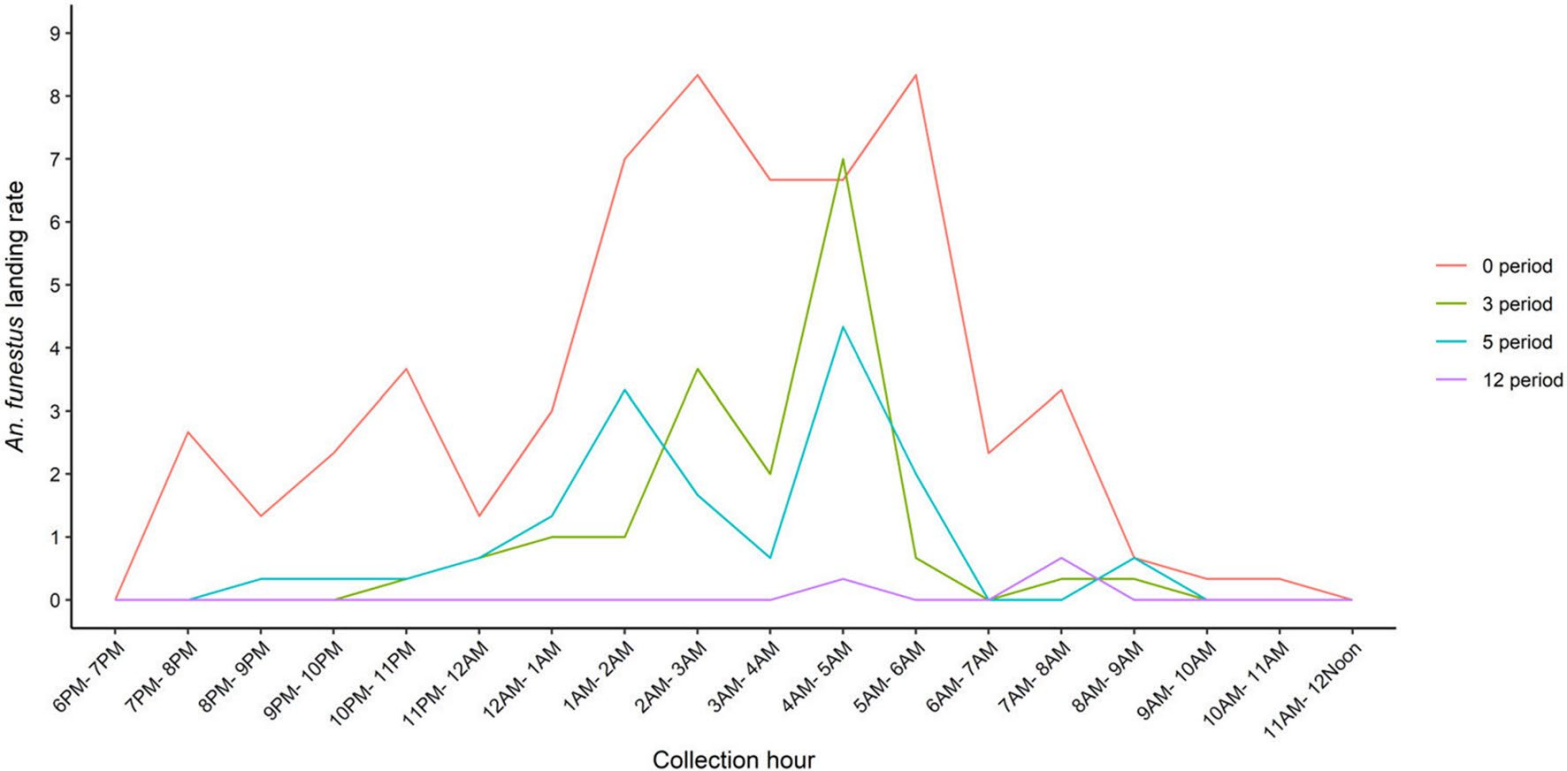
Host-seeking pattern of *An. funestus* in the experimental huts under different emanation periods. Emanation of 3 h indicates emanation between 18:00 and 20:00 and between 05:00 and 06:00, 5 h indicates emanation between 18:00 and 22:00 and between 05:00 and 06:00, and 12 h indicates emanation between 18:00 and 06:00 non-stop

The landing rate of *An. funestus* was observed to be significantly lower in hut with a 12-h emanation period (odds ratio [OR] = 0.14; 95% CI [0.004–0.050]; *P* < 0.0001) when compared to control. However, there was no significant difference in landing rates in the hut with a 2-h emanation period (OR = 0.941; 95% CI [0.518–1.709]; *P* = 0.8418) compared with the control hut (Table 3). Adding an extra hour of emanation between 05:00 and 06:00 to the 2- and 4-h emanation periods resulted in a significant decrease in the landing rate of *An. funestus* in experimental huts. This reduction was observed in huts with a 3-h emanation period (RR = 0.195; 95% CI [0.104–0.364]; *P* < 0.0001) and 5-h emanation period (RR = 0.208; 95% CI [0.112–0.384]; *P* < 0.0001) compared with the 2-h and 4-h emanation periods, respectively (Table 3). The host-seeking trend of *An. funestus* in the experimental huts demonstrated a distinct bimodal pattern, with the first increase in mosquito activity occurring between 02:00 and 03:00, followed by a second peak between 05:00 and 06:00 (Fig. 3).

**Table 3:** Mean landing rates of *An. funestus* in treated and untreated huts under different emanation periods

Emanation period (h)	Mean	Relative risk	*P*-value
12	0.056	0.014 (0.004–0.050)	< 0.0001
5	0.870	0.208 (0.112–0.384)	< 0.0001
4	3.775	0.566 (0.319–1.002)	0.0507
3	0.944	0.195 (0.104–0.364)	< 0.0001
2	6.281	0.941 (0.518–1.709)	0.8418
0	6.675	Ref.	

Odds ratio, 95% confidence intervals and *P*-values were obtained from statistical analysis using generalized linear models. Data from the emanation periods were aggregated; all emanation periods were compared with the negative control

### Outdoor evaluation of metofluthrin SR

**Fig. 4:**
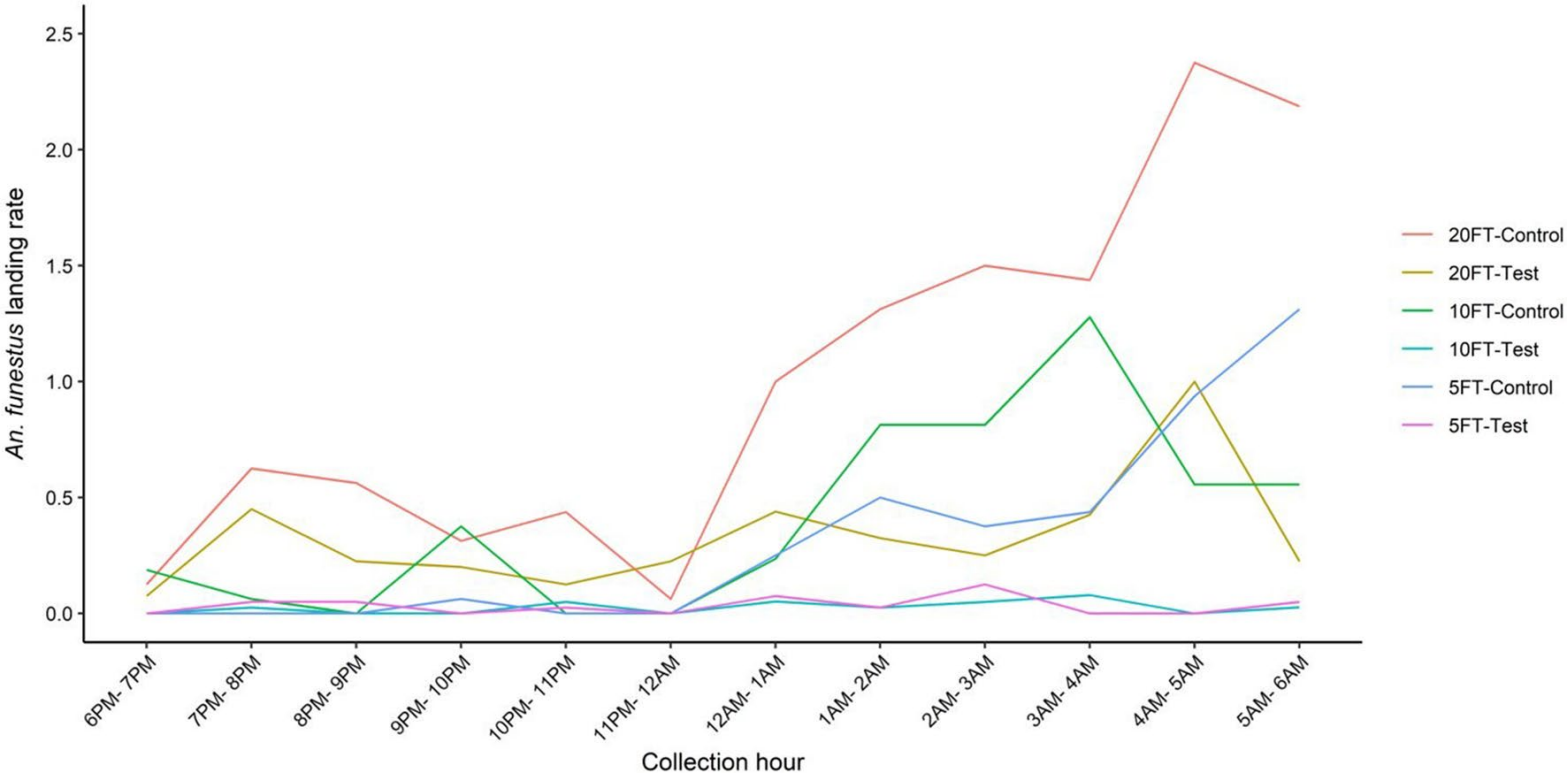
Outdoor host-seeking pattern of *An. funestus* in the villages at emanation distances of 5, 10 and 20 feet

The mean landing rate of *An. funestus* mosquitoes per emanation distance is presented in Table 4. The mean landing rate of *An. funestus* in the presence of metofluthrin SR was significantly lower at all emanation distances than when metofluthrin SR was absent: 5 feet (RR = 0.151; 95% CI [0.070–0.327]; *P* < 0.001), 10 feet (RR = 0.063; 95% CI [0.021–0.192]; *P* < 0.001) and 20 feet (RR = 0.547; 95% CI [0.331–0.905]; *P* = 0.019). The outdoor host-seeking pattern exhibited two peaks in landing activity as shown in Fig. 4.

**Table 4:** Comparison of mean landing rate of *An. funestus* outdoors in the presence and absence of metofluthrin SR at varying emanation distances

Emanation distance	Metofluthrin SR	Mean	Relative risk	*P*-value
5 feet	Yes	0.034	0.151 (0.070–0.327)	< 0.001
	No	0.323	Ref.	
10 feet	Yes	0.025	0.063 (0.021–0.192)	< 0.001
	No	0.417	Ref.	
20 feet	Yes	0.331	0.547 (0.331–0.905)	0.019
	No	0.995	Ref.	

Odds ratio, 95% confidence intervals and *P*-values were obtained from statistical analysis using generalized linear models. Data from the emanation distances were aggregated; all treatments were compared with the negative control

## Discussion

The deployment of metofluthrin SR in the experimental huts resulted in reduced entry, reduced biting and high knockdown and mortality of *An. funestus* within 24 h. Our study showed over 99% reduction in landing rates of *An. funestus* when metofluthrin emanators were placed indoors for 12 h. However, marginal variations were witnessed at reduced emanation periods (2-h and 4-h), and mosquito numbers increased within the next hour, indicating that the SR had no residual effect and thus may need to be paired with LLINs as a complementary tool when people go to bed [26]. In this experiment, additional coverage was considered by the addition of a 1-h period of emanation in the morning between 05:00 and 06:00 when mosquitoes have been shown to be most active and as people wake up and leave, their bednets [27]. The 2–4-h emanation at night would protect individuals inside the house before they go to sleep, where they would be under their bednets, and the additional hour in the morning would prevent biting when people are likely to be waking up and around the house before they leave for the day. This study, therefore, proposes targeted emanation periods for this product given the proposal to attach it to LPG cookers where it would passively emanate as people cooked their dinner and breakfast, respectively. Metofluthrin SR has shown potential for reducing human–mosquito contact [17, 28, 29], and here we demonstrate that an LPG-dependent emanator could be an effective complement to ensure reduced human–vector contact indoors when people are not under their LLINs.

In comparison with *An. arabiensis*, *An. funestus* demonstrated a distinct bimodal pattern in host-seeking both indoors and outdoors, with the first increase in mosquito activity occurring between 02:00 and 03:00, which is a time when the majority of people would be asleep under their LLINs, as reported previously [30, 31], followed by a second peak between 05:00 and 06:00. There was still a high level of mosquito activity even after the collections had stopped, suggesting that it may be necessary to conduct HLC collections in the late morning hours to monitor the behaviour of vectors. The second peak of mosquito activity occurred when most people were out of the protection of LLINs, and hence the need for additional vector control strategies like SRs. The high density of *An. funestus* reported in this study and others [32–34] in western Kenya indicates that *An. funestus* is the dominant malaria vector both indoors and outdoors, with an early morning peak in biting indicating potential biting when people are just stepping away from the cover of their LLINs. Thus, LLINs alone will not be sufficient for malaria vector control. The decline in the *An. gambiae *s.l. population in the study area could be because of LLIN use, as indicated in previous works [33, 35], or attributable to differences in their breeding preference, especially given that this study was conducted in the dry season when abundant *An. funestus* density was due to the swamps on the edge of Lake Kanyaboli.

Metofluthrin significantly reduced the *An. funestus* landing rate. However, its effectiveness decreased with increasing distance from the emanator (85% at 5 feet, 94% at 10 feet, and 45% at 20 feet). This variation in outdoor protective efficacy could be due to the effect of wind, as reported previously [36]. In addition, the technology lacks a residual effect, has high operational costs and requires a configuration that combines an emanator and cooker. This study confirms the protective efficacy of metofluthrin SR against mosquito bites, as reported in other studies [37], and in this case shows an additional benefit in outdoor use, indicating that SRs could play a role in both indoor and outdoor control of mosquitoes and could therefore be deployed in response to changing mosquito behaviour [38].

This study revealed increased entry of *An. funestus* mosquitoes into the huts with LPG cookers relative to the control hut, indicating the added attraction of mosquitoes to houses where LPG cookers are used. Previous experimental and observational studies assessing the combustion effects of LPG on mosquito behaviour in houses have reported similar effects of LPG on *Anopheles* mosquito behaviour [21, 39]. As LPG is projected to become the dominant fuel in many malaria-endemic countries [40], we must consider the fact that indoor LPG use is likely to increase exposure to *Anopheles* through increased household entry and host-seeking [41–43] due to increased production of carbon dioxide (CO_2_) per kilogram of fuel than commonly used fuels such as charcoal and wood [44, 45]. An increase as small as 0.01% in ambient CO_2_ levels above baseline levels can stimulate female mosquitoes to search for blood meals [20, 46] and could explain the increased abundance. Vector control tools that can be coupled with LPG stoves therefore could provide the additional benefit of reduced malaria transmission in Africa.

## Conclusions

The deployment of the metofluthrin-based SR indoors almost completely prevented *An. funestus* landing indoors and led to 10 times lower biting rates within 10 feet of the emanator outdoors. The effectiveness of Thermacell^®^-based metofluthrin SRs certainly warrants their inclusion in the package of vector control tools aimed at reducing human–mosquito contact both indoors and outdoors. The observation of higher mosquito numbers with the use of LPG gas indoors suggests that cooking with LPG can potentially increase human–mosquito exposure to *Anopheles* mosquitoes, hence increasing malaria transmission.

## Data Availability

All the raw data are available and were submitted alongside this manuscript.
